# Determinants of household’s dietary diversity during the COVID-19 pandemic: A community-based study in rural Southwestern Bangladesh

**DOI:** 10.1371/journal.pone.0322894

**Published:** 2025-05-06

**Authors:** Suvasish Das Shuvo, Tamanna Aktar, Asma Khatun, Md. Mohtasim Hasan, Tapon Kumar Das, Md Emran Hossain, Md. Sakhawot Hossain

**Affiliations:** 1 School of Medical, Indigenous, and Health Sciences, University of Wollongong, Wollongong, New South Wales, Australia; 2 Department of Nutrition and Food Technology, Jashore University of Science and Technology, Jashore, Bangladesh; Federal University of Agriculture Abeokuta, NIGERIA

## Abstract

**Background:**

Inadequate dietary diversity is a significant challenge in public health for low-and middle-income countries, including rural communities in Bangladesh. These issues have intensified and become more tragic during the COVID-19 pandemic. This study evaluated the factors associated with household dietary diversity in rural Southwestern Bangladesh during the COVID-19 pandemic.

**Methods:**

This cross-sectional study used a structured questionnaire to collect data from 310 respondents using face-to-face interviews. Household Dietary Diversity Score (HDDS) and sociodemographic characteristics were calculated from the Food and Nutrition Technical Assistance III Project (FANTA) guidelines and related studies. A multinomial regression model was performed to identify factors associated with HDDS during the COVID-19 outbreak.

**Result:**

The HDDS status of rural Southwestern households decreased (60.3%) during the initial COVID-19 pandemic. Socioeconomic factors including gender, level of education, occupation, household monthly income, and family size of the household head were significantly associated with dietary diversity. Additionally, income condition (RRR:5.46, 95% CI:2.73–7.47 and RRR:4.85, 95% CI:2.48–7.24), and dietary diversity knowledge of the household head (RRR:5.46, 95% CI:2.73–7.47, and RRR:4.85, 95% CI:2.48–7.24) were significantly associated with low and moderate HDDS during the COVID-19 pandemic.

**Conclusion:**

This study found that households become more vulnerable to inadequate dietary diversity due to poor socioeconomic status during the COVID-19 pandemic. Based on the findings, public health workers should ensure adequate food access and proper food distribution among rural households and communities in this crisis to mitigate these negative consequences.

## 1. Introduction

Dietary diversity (DD) is considered a global public health issue at national, regional, and household levels where food security is needed through diet diversification [1]. During the COVID-19 pandemic, it was also insufficient in several lower and middle-income countries (LMICs) which poses a challenge for households to maintain a healthy and varied diet [2,3]. Globally, more than 2 billion people still lack access to basic micronutrients, endangering their health and shortening their life expectancy [4]. According to the World Food Program, 1 out of 9 people now do not have enough to eat. Beyond that, over 150 million people experienced extreme poverty and food insecurity worldwide during the pandemic [5]. The majority of people approximately 33 million may have experienced acute food insecurity in Southeast Asia and Sub-Saharan Africa [6]. In Bangladesh, an additional 16.4 million individuals (of whom 12.7 million are from rural regions) are anticipated to live in extreme poverty [7]. In a study conducted in rural Southwest Bangladesh, roughly 80% of the world’s most impoverished and food-insecure individuals reside in remote rural areas [8].

Three pillars of food security, namely availability, access, and utilization, are positively correlated with household dietary diversity [9,10]. Thus, according to UNICEF’s conceptual framework of malnutrition states that due to inadequate dietary intake leads to household food insecurity [11]. It is also regarded that in rural Bangladesh, people’s diets are based on only starchy staples (e.g., rice, tubers, and roots) with insufficient animal products, fruits, and vegetables [12,13]. They are more vulnerable than other households because of limited availability to food, ignorance of varied dietary composition, cultural norms, lower income, and poverty [14,15]. Additionally, following the start of the COVID-19 epidemic, a multifaceted number of causes led to disruptions in global food supply networks and uneven increase in food prices [16]. Movement restrictions, market closures, interruptions to the food market, decreased food production, and other socioeconomic problems contribute to the high prevalence of household food and nutrition insecurity during the COVID-19 pandemic [6,17,18]. As a result, households have experienced a double disaster that has made them more susceptible to food insecurity and malnutrition [19].

Evidence suggests that the COVID-19 epidemic has increased food insecurity and decreased dietary diversity in Mexico, India, Ethiopia, and Nigeria [20–22]. Some recent studies also reported that the COVID-19 pandemic also negatively affects household dietary diversity and overall eating habits [23,24]. Its potential drawbacks include increased mental stress, health services such as impairing physical capacity, lowering immunity, and raising disease susceptibility in rural communities [25–27]. Additionally, these dietary repercussions could create serious health-related comorbidities and difficulties among vulnerable rural households [28]. A high dietary diversity score is related to an increased intake of adequate nutrients from diversified food groups whereas low nutritional diversity is the insufficient nutrient in the diet [29]. However, a study before COVID-19 shows that household food item consumption was reduced in 2020 compared to 2018, with the Household Dietary Diversity Score (HDDS) dropping by 0.44 food categories, from 6.69 to 6.25, among coastal farmers in southern Bangladesh [30]. On top of that, an Asian study on dietary diversity among Chinese residents concluded that people living in areas with a high number of confirmed COVID-19 cases had a lower score of HDD [31]. So, consuming diversified food items is vital for high-quality diets that meet the essential nutrients and important non-nutritive factors and promote good health [29,32].

In Bangladesh, most studies focused on exploring household dietary diversity and food insecurity among children and women during the COVID-19 pandemic [33–36]. To the contextual concern, in Southwestern rural Bangladesh, few studies have been conducted on household dietary diversity status during the COVID-19 pandemic [3,28]. This study focuses specifically on the rural Southwestern regions of Bangladesh, which are uniquely characterized by vulnerabilities such as limited access to markets, reliance on local food production, and recurrent natural disasters like cyclones. By adopting a community-based approach, this research aimed to evaluate the determinants of household dietary diversity in rural Southwestern Bangladesh during the COVID-19 pandemic. The study fills a critical research gap by contextualizing dietary diversity determinants within a specific rural region disproportionately affected by climate and health crises. The findings aim to inform targeted interventions and policy recommendations, addressing the intersection of food security, public health, and disaster resilience in rural Southwestern Bangladesh. This study highlights the importance of localized strategies to enhance dietary diversity and build resilient food systems.

## 2. Methods

### 2.1 Study design and sampling procedure

**Fig 1:**
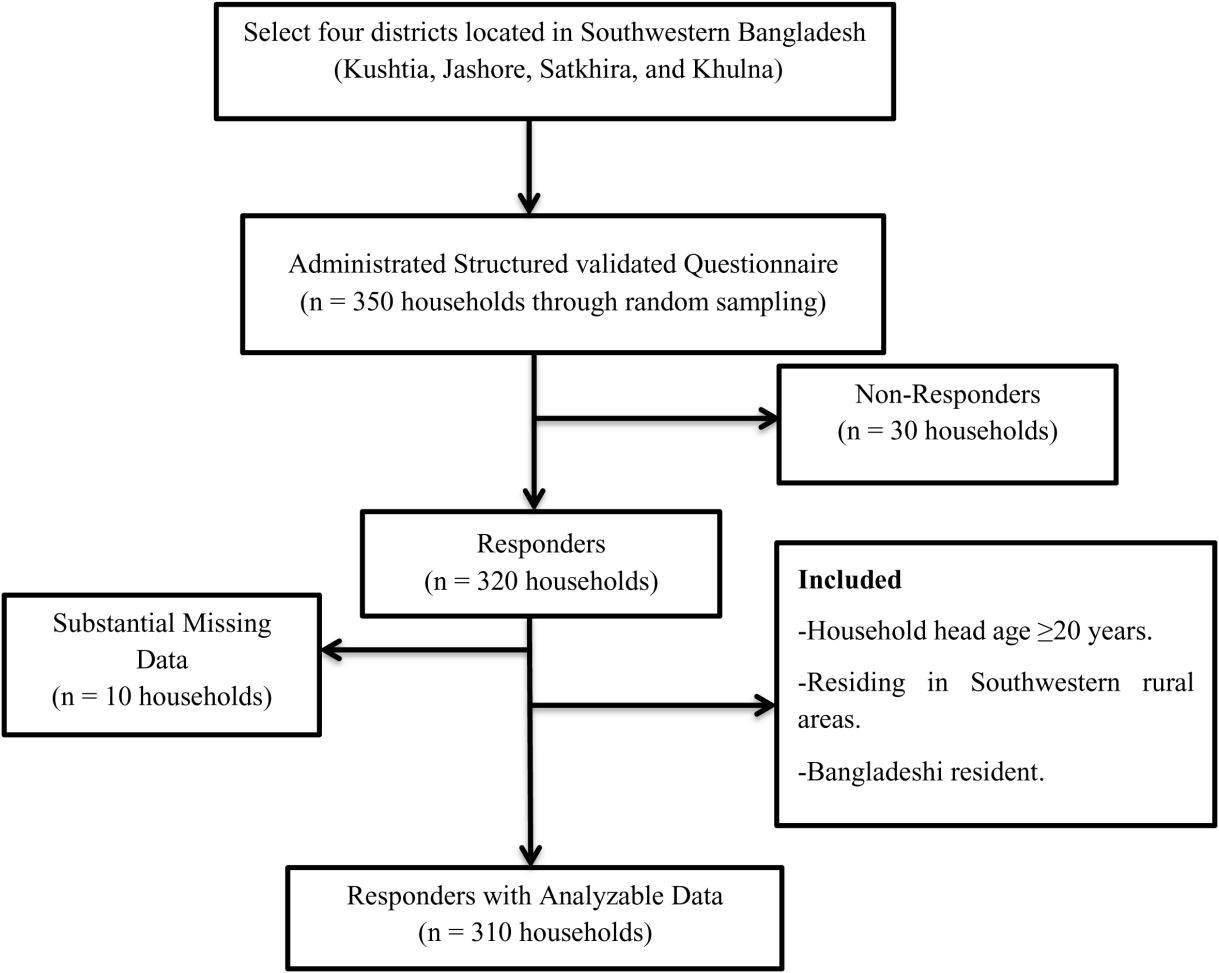
Flow chart of household head recruitment.

A community-based cross-sectional study was conducted in the Southwestern rural areas of Bangladesh, from November 7 to December 27, 2021. A simple random sampling technique was used for sample selection. According to BBS (2011), southwestern rural regions contain 571 unions, 9289 villages, and 3072496 rural households [37]. Among all the districts in the southwestern region, initially, a simple random sampling method was utilized to select four districts located in Southwestern Bangladesh: Kushtia, Jashore, Satkhira, and Khulna. Secondly, four Upazilas in the Southwestern areas were randomly selected. Thirdly, from the four upazillas, eight unions were chosen. The estimated sample size of 310 was determined by using the formula Z^2^p(1-p)/d^2^ with the previously published study’s prevalence of household dietary diversity [38], considering 36% prevalence, 95% of confidence level, 5.4% of margin error, 5% of desired precision, and 20% of non-response rate. Hence, N = Z^2^p(1-p)/d^2^ = (1.96)^2^ × (0.36) × (1–0.36)/(0.054)^2^ ≅ 303. Finally, 310 households were randomly selected for the study (Fig 1). The inclusion criteria included only the head of each household present on the day of the survey, those from a rural area, and in terms of age (i.e., ≥20 years).

### 2.2 Data collection and tools

A structurally validated questionnaire was used to collect data on socioeconomic characteristics and household dietary diversity scores amid the COVID-19 pandemic. The questionnaire was first formatted in English and then translated into Bangla. After completing the survey, bilingual experts retranslated the questionnaire back into English. The purpose of the study was explained to each study participant before the data collection. We conducted a risk assessment to determine the feasibility and safety of conducting face-to-face data collection activities during the pandemic. Additionally, we developed protocols and guidelines for data collectors to adhere to. Furthermore, we recruited four interviewers who received thorough training in COVID-19 prevention measures, encompassing the correct use of masks, gloves, personal protective equipment (PPE), hand sanitizer, and the maintenance of physical distancing during interactions. Informed consent forms were obtained from all household representatives who agreed to participate in the study and signed or applied thumbprints (in ink) were also obtained.

### 2.3 Questionnaire design and variables

Questionnaire was consisted of two sections including socio-economic characteristics and dietary assessment.

#### 2.3.1 Outcome variable.

The main outcome variable of the present study is the Household Dietary Diversity Score (HDDS). HDDS was defined as the food items consumed within 24 hours across 12 distinct food groups (i.e., cereals, roots and tubers, legumes and pulse, fats and oils, meat and poultry, fish, eggs, vegetables, fruits, milk/dairy product, sugar, and other sweeteners, and other foods, such as condiments, coffee, tea) that are used for the assessment of an individual or household dietary diversity. The HDDS questionnaire was developed according to a document from the Food and Nutrition Technical Assistance Project (FANTA), USAID guidelines [39]. One point was given to each food group consumed and a zero score was awarded to a household that did not consume over the reference period. The HDDS scales were calculated both as continuous and categorical variables for analysis. The score range was 0–12, which were categorized into low HDDS (≤3 scores), moderate HDDS (4–6 scores), and high HDDS (≥7 scores) for independent analysis [29,39].

#### 2.3.2 Predictor variables.

The main primary predictor variables were gender (male, female) of the household head, age (20 years and above), level of education (illiterate, primary, secondary, and higher secondary), occupation (farmer/day labor, maidservant, worker, vendor, and job holder), family income per month in Bangladeshi Taka (<5000BDT and above), family member (2 and above), knowledge about a balanced diet (yes and no) and earned less income during COVID-19 than pre-pandemic (yes and no).

### 2.4 Data management and statistical analysis

Data were checked and edited for completeness and accuracy before being entered for analysis. All analyses were done using the STATA statistical software package, Version 14. Standard descriptive statistics were calculated in frequency (n) and percentage (%) for categorical variables. The chi-square test was used to calculate significant differences for categorical variables. Multinomial logistic regression was used to estimate the factors influencing household dietary diversity scores during the COVID-19 pandemic in rural southwestern Bangladesh. Household Dietary Diversity Score status was the main outcome variable and socioeconomic factors were indicated as explanatory variables. The final model incorporates all explanatory variables such as gender, age, education level, household head’s occupation, family income, family size, and income conditions during the COVID-19 pandemic for adjustment. A relative risk ratio with a 95% confidence interval was determined to evaluate the presence and strength of the relationship. P-value < 0.05 was declared for the determination of statistical significance.

### 2.5 Ethics approval

Ethical approval and prior permission were obtained from the institutional Ethical Review Committee before the commencement of the study. Ethical approval for the study was obtained from the Jashore University of Science and Technology Institutional Review Board (Ref: ERC/FBST/JUST/2021–55). Meetings with household representatives were held, the aims and procedure of the study were explained, and written informed consent forms were obtained.

## 3. Results

### 3.1 Socioeconomic characteristics of the households

Table 1 presents that the majority (64.8%) of household heads were males, while 36.5% of respondents were aged between 40–50 years. Concerning education, 44.8% of respondents were illiterate, and only 16.8% had received higher secondary education. In terms of household heads’ occupation; 32.3%, 28.7%, and 30.6% of respondents were farmers, maidservants, and vendors/hawkers respectively. The result also reports that the majority (62%) of household heads’ incomes were below 5000 BDT per month whereas only 5.2% had above 20000 BDT per month. Predominantly, more than three-fourths of the respondents (87.3%) had poor income during the COVID-19 pandemic, and about three-fourths of the respondents (72.6%) did not know about a balanced diet.

**Table 1:** Characteristics of respondents based on household dietary diversity score during the COVID-19 pandemic.

Household’s head traits	Category	Frequency (%)			
		Total	Very good	Moderate	P-value
**Gender**	Male	201 (64.8)	28 (13.9)	69 (34.3)	0.001
	Female	109 (35.2)	3 (2.7)	24 (22.0)	
**Age**	20-30 years	64 (20.6)	3 (4.7)	21 (32.8)	0.35
	30-40 years	103 (33.2)	10 (9.7)	34 (33.0)	
	40-50 years	113 (36.5)	12 (10.6)	32 (28.3)	
	>50 years	30 (9.7)	6 (20.0)	6 (20.0)	
**Level of education**	Higher Secondary	52 (16.8)	34 (65.4)	7 (13.5)	<0.001
	Illiterate	139 (44.8)	8 (5.7)	39 (28.1)	
	Primary	93 (30.0)	3 (3.3)	37 (39.8)	
	Secondary	26 (8.4)	9 (34.6)	10 (38.5)	
**Occupation**	Farmer	100 (32.3)	8 (8.0)	41 (41.0)	<0.001
	Maidservant	89 (28.7)	3 (3.4)	16 (18.0)	
	Worker	12 (3.8)	4 (33.3)	7 (58.4)	
	Vendors/hawkers	95 (30.6)	13 (13.7)	28 (29.5)	
	Job holder	14 (4.6)	10 (71.5)	3 (21.4)	
**Family income per month**	<5000BDT	192 (61.9)	9 (4.7)	63 (32.8)	0.002
	5000-10000BDT	73 (23.5)	12 (16.4)	22 (30.1)	
	10000-15000BDT	29 (9.4)	7 (24.1)	7 (24.1)	
	>20000BDT	16 (5.2)	12 (75.0)	3 (18.7)	
**Family member**	2-4	78 (25.2)	10 (12.9)	24 (30.8)	0.21
	5-8	164 (52.9)	10 (6.2)	49 (29.5)	
	≥8	68 (21.9)	10 (14.7)	20 (29.4)	
**Knowledge about a balanced diet**	Yes	85 (27.4)	16 (18.8)	38 (44.7)	<0.001
	No	225 (72.6)	21 (9.4)	83 (36.8)	
**Earned less Income during COVID-19 than pre-pandemic**	Yes	271 (87.3)	25 (9.2)	78 (28.6)	0.03
	No	39 (12.7)	21 (55.7)	12 (31.5)	

**Note:** HDDS: Household Dietary Diversity; BDT: Bangladeshi Taka, 1 USD ~ 110 BDT.

### 3.2 The prevalence of HDDS regarding socioeconomic characteristics of the respondents

**Fig 2:**
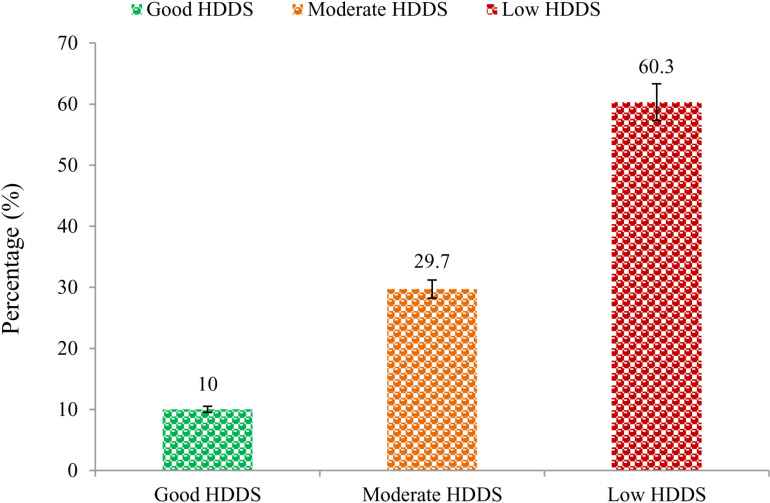
Household Dietary Diversity Status during COVID-19.

The prevalence of HDDS is provided in Fig 2. More than half of the respondent households (60.3%) had low HDDS whereas 29.7% and 10% were categorized as moderate HDDS and good HDDS, respectively. Characteristics of respondents based on household dietary diversity scores during the COVID-19 pandemic as shown in Table 1. The result also shows that 75.3% of female and 51.8% of male household heads had low HDDS while only 2.7% of females had good HDDS. Nearly two-thirds of the illiterate (66.2%) and primary level of education (56.9%) household heads had low HDDS. Based on the household head occupation, the result reported that household heads who were farmers, maidservants, and vendors/hawkers had 51%, 78.6%, and 56.8% low HDDS, respectively. Regarding household income <5000 BDT, nearly 33% and 62.5% of household heads had moderate and low HDDS. In terms of knowledge about a balanced diet, almost 37% and 54% had moderate and low HDDS. Finally, the household heads that had poor income (62.2%) during the COVID-19 pandemic and monthly income of less than 5000 BDT (62.5%) had low HDDS.

### 3.3 Factors associated with household dietary diversity

The multinomial regression analysis explains the factors that are associated with household dietary diversity during the COVID-19 pandemic (Table 2). Female household heads were 4.77 times (95% CI: 2.43–7.16, p < 0.05) and 3.62 times (95% CI: 1.32–5.94, p < 0.05) more likely to have low HDDS and moderate HDDS relative to male counterparts. Respondents aged between 30–40 years were at significantly lower risk of having low HDDS and moderate HDDS relative to others. The result shows that household heads who had primary education were 3.25 times and 2.78 times more likely to have low HDDS and moderate HDDS than higher secondary household heads. Fuethermore, the risk of having low HDDS and moderate HDDS were 4.95 times and 3.45 times more likely in illiterate household heads than educated household heads. The regression analysis further elucidates a significant association between the occupation of household heads and both low and moderate HDDS. Maidservant household heads were 4.61 times and 3.83 times more likely to have low HDDS and moderate HDDS than job holders. Again, the vendors/hawkers had 3.22 times and 3.56 times significantly increased risk of being low in HDDS and moderate HDDS than reference household heads (p < 0.01). Additionally, monthly family income was also associated with moderate and low dietary diversity scores. During the COVID-19 pandemic, households with a monthly income of less than 5000 BDT had 4.65 times and 3.74 times greater likelihood of having low HDDS and moderate HDDS than those with a monthly income of more than 15000 BDT, respectively. The result of this study also shows that the risk of low HDDS and moderate HDDS were 4.16 times and 3.93 times higher in households that had 5–7 members relative to the ≥8 family member counterparts. Moreover, income conditions during the pandemic exhibited a significant association with low HDDS and moderate HDDS (p < 0.01). The respondents who did not know the balanced diet were 5.17 times and 3.41 times more likely to have low HDDS and moderate HDDS (p < 0.01). Household heads who had earned less income during COVID-19 than pre-pandemic were 5.46 times and 4.85 times higher risk of having low HDDS and moderate HDDS than usual-income peers.

**Table 2:** Determinants of household dietary diversity score.

Household’s head trait	Reference: Very good DDS	
	Moderate HDDS	Low HDDS
	RRR (95%CI)	RRR (95%CI)
Gender (reference category: Male)		
Female	**3.62 (1.32-5.94)** ^**^	**4.77 (2.43-7.16)** ^**^
Age (reference category: 20–30 years)		
30-40 years	**0.47 (0.54-0.87)** ^***^	**0.21 (0.14-0.34)** ^**^
40-50 years	0.65 (0.24-1.68)	0.36 (0.26-1.52)
>50 years	0.25 (0.18-1.45)	0.19 (0.08-1.34)
Education level (reference category: higher secondary)		
Illiterate	**3.45 (1.32-5.37)** ^***^	**4.95 (2.67-7.24)** ^***^
Primary	**2.78 (1.24-4.38)** ^**^	**3.25 (1.43-5.13)** ^**^
Secondary	0.75 (0.24-1.27)	0.92 (0.42-1.46)
Occupation (reference category: Job holders)		
Farmer	1.68 (0.63-2.79)	1.21 (0.65-1.80)
Maidservant	**3.83 (1.76-5.89)** ^**^	**4.61 (2.11-7.17)** ^***^
Worker	2.21 (0.13-3.34)	2.75 (0.16-4.36)
Vendors/hawkers	**3.56 (2.04-5.12)** ^***^	**3.22 (1.32-5.14)** ^**^
Family income per month (reference category: above 15000BDT)		
<5000 BDT	**3.74 (1.44-6.18)** ^***^	**4.65 (2.46-6.87)** ^***^
5000-10000 BDT	2.86 (0.16-4.67)	3.74 (0.76-5.81)
10000-15000 BDT	1.16 (0.35-1.06)	1.28 (0.53-2.09)
Family member (reference category: 2–4)		
5-7	**3.93 (2.02-7.23)** ^**^	**4.16 (2.27-6.08)** ^**^
≥8	3.97 (0.75-6.23)	4.01 (0.65-5.38)
Knowledge about a balanced diet (reference category: Yes)		
No	**3.41 (2.15-4.67)** ^***^	**5.17 (2.61-7.63)** ^***^
Earned less Income during COVID-19 than pre-pandemic (reference category: No)		
Yes	**4.85 (2.48-7.24)** ^***^	**5.46 (2.73-7.47)** ^***^

**Note:**

***p ≤ 0.01,

**p ≤ 0.05; HDDS: Household Dietary Diversity; BDT: Bangladeshi Taka, 1 USD ~ 110 BDT.

## 4. Discussion

The emergence of the COVID-19 pandemic has generated an additional crisis for vulnerable households, especially among rural communities. Concerning this issue, the present study was to explore the household dietary diversity patterns and their associated factors in the rural community in Southwestern areas of Bangladesh during the COVID-19 pandemic. We found that more than half of the households had low dietary diversity (60.3%). This finding is consistent with other evidence found in different settings [3,40-41]. This could be due to inadequate food consumption which is linked with less income and increased food prices during COVID-19 [27,42]. However, low socioeconomic conditions among rural households may also have made it difficult to purchase protein and micronutrient-rich food during the pandemic situation. The results of this study revealed that several socio-economic factors were associated with household dietary diversity during the COVID-19 pandemic in the Southwestern regions of Bangladesh. Our study showed that households headed by females had moderate and lower HDDS than male-headed households during the COVID-19 pandemic. Similar results are found in earlier studies conducted in Ethiopian and Iranian rural households [2,43]. It may be regarded that female-headed households faced enormous issues including lower income, limited access to capital, land ownership, market, and new technologies in rural households during the pandemic [44,45].

Importantly, the income crisis generated during this COVID-19 pandemic greatly affected household diets. We identified that households aged between 30–50 years were more likely to have moderate and low HDDS than their counterparts. Our study is consistent with the previous study conducted in Ecuador [40]. This might be a result of the COVID-19 pandemic condition’s reduced employment opportunities and the disabilities of individuals without the means to purchase food [46]. The findings of the study further observed that illiterate household heads were significantly associated with lower HDDS than higher secondary education household respondents. The findings of this study are consistent with other studies conducted in Ethiopia, Tanzania, and India [2,27,47]. This is probably because illiteracy leads to lower opportunities for employment and also decreases household dietary diversity [48]. In the current study, maidservants, and vendors/hawker’s household heads had a significantly higher risk of having moderate HDDS and low HDDS than job-holder-headed households. Other studies revealed that employment status was significantly associated with household dietary diversity [49–51]. Most businesses and jobs were shut down due to COVID-19, and people were not permitted to continue their income-generating activities [52].

Our study also reported that household heads with poor income and family incomes below 5000 BDT per month were significantly associated with moderate HDDS and low HDDS. Several consistent studies have been conducted in Bangladesh, Ecuador, and Namibia [49–51]. This could be due to the restriction of movement; most people become jobless while poor households may restrict their food intake to an insufficient amount during the COVID-19 pandemic [19]. The current investigation confirmed that households with family sizes of 5–7 were more likely to have moderate HDDS and low HDDS than households with family sizes of 2–4. Other recent studies are also consistent with this finding [27,50]. There may be regard to decreasing income and increasing food prices with large family members during the COVID-19 lockdown [28]. Moreover, our study also stated that inadequate knowledge about a balanced diet was significantly associated with lower HDDS among the household respondents. Similar results are obtained in prior studies conducted in Bangladesh, Ethiopia, and Tehran [28,52,53]. One possible explanation for this finding could be due to inadequate nutrition education and a lack of awareness of making healthy dietary choices, maintaining optimal health, and reducing risks of diseases [54].

Most low and middle-income countries confronted different social, economic, and political challenges to manage the disastrous problems during the COVID-19 pandemic. Recognizing the socio-economic impact of the COVID-19 pandemic on households’ dietary diversity may support governments, NGOs, health experts, and policymakers in coping with the different detrimental effects on people’s quality of life. Several food aid programs by the government can contribute to improving household dietary diversity status due to closing most occupations, increasing the rate of unemployment, and potentially decreasing the economic situation over time during this pandemic. Besides, different social media may play a significant role in increasing the importance of dietary diversity-related knowledge.

This study has some limitations. First, the cross-sectional design prevents drawing any causal inferences between food dietary diversity and its predictors. Second, although DDS provides a suitable proxy for household food consumption, it does not reliably predict whether consumed food meets the required dietary intake. Third, household heads’ self-reported dietary diversity, socioeconomic characteristics, and recall bias may arise. Additionally, due to the short study duration, it was not possible to assess the seasonal variation of dietary diversity among the participants. Finally, in this study, it was not possible to collect a large amount of data by face-to-face interviews due to movement restrictions during this pandemic. Despite these limitations, one of the strengths of this study is that it is the first of its kind to assess the impact of COVID-19 on household dietary diversity among Southwestern rural areas in Bangladesh. Another strength was that the data were collected through the face-to-face survey, which helped to choose the target population correctly with the included questions. Overall, this is a community-based cross-sectional study that provides a snapshot of dietary diversity in rural Southwestern areas of Bangladesh.

## 5. Conclusion

In summary, during the COVID-19 pandemic, the prevalence of low HDDS was high in rural Southwestern regions of Bangladesh. The finding also showed that gender, level of education, occupation of household head, household head income, family size, and knowledge about a balanced diet were the main contributing factors to low HDDS among them. However, this study emphasizes how critical it is to offer sufficient assistance to these most vulnerable populations in meeting their basic needs. Due to the negative impacts of the COVID-19 pandemic, as well as the necessity of nutrition education, raising awareness, financial support, and creating income-generating sources etc. among rural households. Finally, it will be necessary that the government and policymakers recognize the negative impact of poor DDS and take strategies to mitigate the adverse effects of the COVID-19 pandemic on households’ physical and mental health.

## Supporting information

S1 Questionnaire Study questionnaire. (DOCX)
