# Supplementary material for: Determinants of household’s dietary diversity during the COVID-19 pandemic: A community-based study in rural Southwestern Bangladesh
Source: PLoS One. 2025 May 6;20(5):e0322894. doi: 10.1371/journal.pone.0322894 (PMC12054898; doi:10.1371/journal.pone.0322894)
Supplement: S1 Questionnaire — (DOCX) [file pone.0322894.s001.docx]

**Determinants of Household's Dietary Diversity during COVID-19 Pandemic: A Community-based Study in Rural South-Western Bangladesh**

**Questionnaire**

1. **Characteristics of the Households head respondents**

| **Variables** | **Category** |
| --- | --- |
| **Gender** | Male |
|  | Female |
| **Age** | 20-30 years |
|  | 30-40 years |
|  | 40-50 years |
|  | >50 years |
| **Level of education** | Higher secondary |
|  | Illiterate |
|  | Primary |
|  | Secondary |
| **Occupation** | Farmer |
|  | Maidservant |
|  | Worker |
|  | Vendors/hawkers |
|  | Job holder |
| **Family income per month** | <5000BDT |
|  | 5000-10000BDT |
|  | 10000-15000BDT |
|  | >20000BDT |
| **Family member** | 2-4 |
|  | 5-8 |
|  | ≥8 |
| **Knowledge about dietary diversity** | Yes |
|  | No |
| **Income conditions** | Poor |
|  | Usual |

1. **Household Dietary Diversity Score (HDDS) for Measurement of Household Dietary Diversity during the COVID-19 pandemic**

| **HDDS** | **Score (24 hour)** | |
| --- | --- | --- |
|  | **Yes** | **No** |
| Now I would like to ask you about the types of foods that you or anyone else in your household ate yesterday during the day and at night. | | |
| Any cereals like bread, rice noodles, biscuits, or any other foods made from millet, sorghum, maize, rice, wheat, or [insert any other locally available grain]? | 1 | 0 |
| Any roots and tubers? | 1 | 0 |
| Any legumes and pulse? | 1 | 0 |
| Any fats and oils? | 1 | 0 |
| Any meat and poultry? | 1 | 0 |
| Any fish? | 1 | 0 |
| Any eggs? | 1 | 0 |
| Any vegetables? | 1 | 0 |
| Any fruits? | 1 | 0 |
| Any milk/dairy product? | 1 | 0 |
| Any sugar and other sweeteners? | 1 | 0 |
| Any other foods, such as condiments, coffee, tea? | 1 | 0 |

1. **Food Frequency Questionnaire among the households during the COVID-19 pandemic**

| **FFQ Conditions** | **FFQ per week** | | | |
| --- | --- | --- | --- | --- |
|  | **No intake** | **Rarely** | **Sometimes** | **Always** |
| Now I would like to ask you about the types of foods that you or anyone else in your household ate during the last seven days. | | | | |
| Any cereals like bread, rice noodles, biscuits, or any other foods made from millet, sorghum, maize, rice, wheat, or [insert any other locally available grain]? |  |  |  |  |
| Any roots and tubers? |  |  |  |  |
| Any legumes and pulse? |  |  |  |  |
| Any fats and oils? |  |  |  |  |
| Any meat and poultry? |  |  |  |  |
| Any fish? |  |  |  |  |
| Any eggs? |  |  |  |  |
| Any vegetables? |  |  |  |  |
| Any fruits? |  |  |  |  |
| Any milk/dairy product? |  |  |  |  |
| Any sugar and other sweeteners? |  |  |  |  |
| Any other foods, such as condiments, coffee, tea? |  |  |  |  |

**Participant information and informed consent form**

**Ethical review committee of Jashore University of Science and Technology, Jashore, Bangladesh**

**Informed consent form for research involving human subjects**

**Project Title:** Determinants of Household's Dietary Diversity during COVID-19 Pandemic: A Community-based Study in Rural South-Western Bangladesh

**Supervisor/Principal Investigator:** Suvasish Das Shuvo

**Institution:** Jashore University of Science and Technology, Jashore, Bangladesh

**Email:** [shuvo_nft@just.edu.bd](mailto:shuvo_nft@just.edu.bd)

**N.B.:** This is the representative form from the participants of the designated study area. The supervisor could able to provide all participants consent form if Ethical review committee claimed.

**Authorization Statement**

I have read each page of this paper about the study (or it was read to me). I know that being in this study is voluntary and I choose to be in this study. I know I can stop being in this study without penalty. I will get a copy of this consent form now and can get information on results of the study later if I wish.

Participant’s name: Date:

Participant’s signature: Time:

*Consent form explained/witnessed by:

Signature

Printed name:

Date: Time:

(*is the person who has explained the research to the participant/participant’s representative and has answered any question he/she has about the research)
